# *“If government is saying the regulations are important, they should be putting in funding to back it up.”*- An in-depth analysis of local authority officers’ perspectives of the Food (Promotion and Placement) (England) Regulations 2021

**DOI:** 10.1186/s12916-024-03720-5

**Published:** 2024-11-06

**Authors:** Preeti Dhuria, Sarah Muir, Sarah Jenner, Emma Roe, Wendy Lawrence, Janis Baird, Christina Vogel

**Affiliations:** 1 Medical Research Council Lifecourse Epidemiology Centre, University of Southampton, Southampton General Hospital, Tremona Road, Southampton, SO16 6YD UK; 2 School of Psychology, University of Southampton, Highfield Campus, Southampton, SO17 1BJ UK; 3 School of Geography and Environmental Science, University of Southampton, Highfield Campus, Southampton, SO17 1BJ UK; 4 Primary Care, Population Science and Medical Education, Faulty of Medicine, University of Southampton, Highfield Campus, Southampton, SO17 1BJ UK; 5 National Institute for Health Research Southampton Biomedical Research Centre, University of Southampton, and University Hospital Southampton NHS Foundation Trust, Tremona Road, Southampton, SO16 6YD UK; 6 NIHR Applied Research Collaboration Wessex, Southampton Science Park, Innovation Centre, 2 Venture Road, Chilworth, Southampton, SO16 7NP UK; 7 Centre for Food Policy, University of London, City St George’s, Northampton Square, London, EC1V 0HB UK

**Keywords:** Food (Promotion and Placement) regulations, Enforcement, Qualitative analysis, Food environment, Food policy, Local authority officers

## Abstract

**Background:**

As part of the UK government’s obesity strategy, the Food (Promotion and Placement) (England) Regulations 2021 were implemented in October 2022 to restrict the prominent placement of products high in fat, sugar, or salt (HFSS) in most retail settings. Local authority (LA) officers have been tasked with enforcement of these regulations. This qualitative study examined the perspectives of LA officers including, trading standards, environmental health, and public health officers to understand enforcement approaches and requirements to optimise business compliance with the regulations.

**Methods:**

Semi-structured interviews were conducted via MS Teams with a purposive sample of LA officers across England. Data were analysed using inductive thematic analysis.

**Results:**

The 22 participants comprised 13 officers from Trading Standards, six from Environmental Health, and three from Public Health teams. The key messages include the following: (i) the regulations are complex and do not align with existing enforcement approaches, (ii) officers’ professional background will result in variable enforcement practices, and (iii) compliance assessment is an arduous task. LAs are facing resource and workforce constraints and have to prioritise regulations addressing high health risks (e.g., allergens). Therefore, officers will mostly apply a light touch approach to enforcement, raising awareness and engaging with businesses rather than issuing notices. To develop a consistent enforcement approach across LAs, officers asked for (i) further leadership from central government in the form of funding, training, and tools to determine in-scope businesses and products, (ii) cross-departmental collaboration to raise the regulations’ priority at local and regional levels, and (iii) greater consumer demand for healthier retail environments.

**Conclusion:**

It is crucial to address both structural challenges such as resource allocation, workforce, and prioritisation issues as well as the inherent complexity of the regulations to strengthen enforcement efforts. Our findings highlight the necessity of supporting enforcement activities at national and regional government levels to avoid potential false conclusions about ineffectiveness of regulations.

**Supplementary Information:**

The online version contains supplementary material available at 10.1186/s12916-024-03720-5.

## Highlights

• The UK Food (Promotion and Placement) regulations are perceived as complex and low priority by LA officers

• A light touch enforcement approach could undermine effective implementation of the regulations

• Funding is required to enable officers to conduct business support visits and encourage compliance

• Accredited training for officers is required to facilitate common understanding of regulations

• Qualitative methods provided novel insights about enforcement concerns of the pioneering Food (Promotions and Placement) regulations to reduce obesity

**Supplementary Information:**

The online version contains supplementary material available at 10.1186/s12916-024-03720-5.

## Background

### Role of local authorities (LAs) in public health

The LAs in the UK have a key role in improving health of their communities as accorded by the 2012 Health and Social Care Act through encouraging and enforcing compliance of various regulations [1]. A significant piece of legislation, the Food (Promotion and Placement) (England) Regulations 2021 (*hereafter the regulations*), was recently introduced to address the current obesity crisis. The success of these regulations largely depends on their effective implementation and enforcement, with the responsibility for the latter entrusted to LAs. Broadly, there are two types of LAs in England: (i) single tier including unitary councils, London boroughs, and metropolitan boroughs where services are undertaken by one single body and (ii) two-tier authorities incorporating county and district councils who collectively cover responsibility for public services including public health [2]. In England, as of April 2023, there are a total of 317 LAs including 21 county councils, 164 district councils, and 132 unitary authorities [2]. Within LAs, the responsibility for enforcing various regulations lies with two types of enforcement officers (*hereafter officers*): environmental health officers (EHOs) and trading standards officers (TSOs) [3]. EHOs are usually employed by district and unitary councils, while TSOs are employed by county councils [2, 4]. EHOs focus primarily on monitoring food storage, food safety, and enforcing appropriate food handling practices [5]. In contrast, TSOs focus on protecting consumers from unfair trading practices and enforcing a wide range of regulations, including those related to the sale of goods, product safety, pricing, advertising, and labelling [3, 6]. The specific duties and responsibilities of EHOs and TSOs, however, vary across different LAs depending on local priorities and resources [7]. EHOs and TSOs have different skill sets and expertise. These differences could have implications for how they enforce various regulations [5, 6]. Under a statutory government scheme, many larger businesses enter in a paid primary authority partnership with a single LA to obtain assured regulatory advice tailored to their business on complying with the regulations and offers some protection from other enforcing authorities [8].

### Policy context and the regulations

The UK has the third highest rate of obesity in Europe, largely due to excessive availability, access, and consumption of foods that are energy dense, nutrient poor, highly processed [9, 10]. Unhealthy foods are an obvious choice for businesses to market due to their high palatability, low manufacturing costs, and long shelf life, all of which contribute to substantial profit margins [11]. Moreover, profitability of these items is further enhanced through effective branding and marketing [12]. Businesses often place unhealthy foods in prominent locations to promote impulse purchases of these items [13, 14]. Given these challenges, stronger government intervention was deemed necessary to support consumers in making healthier choices and to address widening dietary inequalities [15–17]. A synthesis of systematic reviews has identified that addressing price, price promotions, and product placement are among the most effective strategies for promoting healthy purchasing in supermarkets [18]. There is moderate evidence that interventions which limit the prominent placement and price reduction strategies of unhealthy items could improve diet-related practices and population diets [19–21]. Increasing evidence on the effectiveness of retail interventions and increasing calls from industry for a level playing field prompted the UK government to enact the regulations in 2021 to reshape unhealthy food marketing practices in retail settings [22]. As per legislative procedure, two public consultations, in 2019 and 2020, were conducted to determine the regulations’ scope, definitions, and enforcement arrangements [23, 24]. These regulations signify a shift from a previous less interventionist policy approach, which relied on voluntary efforts by industry to improve healthier standards or placed responsibility firmly with individuals [25, 26]. Further details of the regulations are provided in Table 1.

**Table 1:** Food (Promotion and Placement) Regulations 2021

**Who is impacted?** ✓ Medium and large businesses (with 50 employees or more) ✓ Stores with a relevant floor area of 2000 sq.ft or more ✓ Online retailers ✓ Non-food retailers (such as DIY stores, pharmacies, and clothes stores) **What is restricted?** ✓ High fat, sugar, and salt (HFSS) **prepacked food and drinks** in **thirteen categories** ✓ HFSS score is defined using nutrient profile model ✓ Volume price promotions on HFSS foods *** e.g. BOGOF, 50% extra free, 3 for 2, 3 for £10 in-store or online*** ✓ Placing HFSS items in prominent locations in store *** e.g. within 2 m of checkout, within 2 m of a designated queuing area, store entrances, aisle ends,*** etc ✓ Placing HFSS items in prominent locations online *** e.g. on homepages, checkout pages, ‘favourites’ products page, pages not opened intentionally by the consumer,*** etc **Who will enforce?** Local authorities ✓ Trading standards officers ✓ Environmental health officers	

Medium and large retailers with more than fifty employees, and with a store area of more than 2000 square feet, which sell prepacked food or drink directly to the public are in scope of these regulations (e.g. supermarkets, large convenience stores, high-street stores that might sell food products). Out-of-home businesses that sell prepared products to be consumed as a set meal (e.g. restaurants, takeaways) and specialist retailers selling mainly one type of product are not included. The placement component restricts the positioning of prepacked HFSS products in high traffic areas including store entrances, aisle ends, and checkouts; online equivalents are also included. In-scope HFSS products from 13 broad categories (namely soft drinks, savoury snacks, breakfast cereals, confectionary, ice cream and lollies, cakes and cupcakes, sweet biscuits and bars, morning goods, desserts and puddings, sweetened yogurt, pizza, potato products, and prepared meals, products in sauce and breaded or battered foods) can no-longer be displayed in these key locations; non-HFSS products, non-food, alcohol, and tobacco products can still be placed at store entrances, aisle ends, and checkouts [27]. A food is classed as HFSS if it scores four points or more, and a drink is classed as HFSS if it scores one point or more on the UK government’s nutrient profiling model (NPM) 2004/05 [28].

The placement component of the regulations commenced implementation in England from 1 October 2022 to prohibit the placement of unhealthy foods in prominent locations; however, the volume promotion component, such as three items for the price of two, has been delayed until October 2025 due to food price inflation during the cost-of-living crisis [22, 29]. Similar regulations are being planned in Scotland and Wales [30, 31]. The responsibility for selling HFSS products in line with this regulations falls upon retailers and manufacturers, but liability from non-compliance falls upon retailers [32].

### Enforcement of the regulations

The regulations grant discretion to LA officers in conducting enforcement investigations, allowing for tailored responses to non-compliant behaviours. These actions could range from raising awareness through to monetary penalties. Enforcement officers are empowered with statutory authority to address regulatory breaches, with the ability to issue improvement notices and escalate to fixed monetary penalty (FMP) of £2500 for continued non-compliance. Retailers can mitigate penalties by paying 50% within 28 days, but failure to do so may result in final notices and criminal proceedings [32].

### Rationale and key research questions

LA officers play a pivotal role in assessing compliance, and their lived experiences can reveal important insights about regulation implementation including unintended consequences, enforcement challenges, and support needs. Their interactions with businesses have the potential to shape regulatory outcomes of the regulations. In LAs, public health officers (PHOs) play a critical role in promoting health and addressing obesity through a range of preventative measures, including working with local businesses to create healthy food environments. PHOs have a vested interest in and subject-knowledge related to the successful implementation of these regulations given their aim to address childhood obesity. PHOs' insights on how these regulations could be prioritised in LAs makes them key stakeholders in this research. Additionally, in some LAs, PHOs assist EHOs and TSOs in interpreting the guidance for the regulations, as they often possess expertise in nutrition. A systematic review of qualitative literature on previous policies and interventions to improve health in food retail settings highlighted limited investigation of the lived experiences of officers with responsibility for assessing compliance and conducting enforcement (*Dhuria *et al*., in draft*). The aim of this study was to explore LA officers’ perspectives on enforcement approaches and requirements to optimise business compliance with the regulations. By incorporating officers’ viewpoints, a more inclusive and participatory approach to the regulations can be achieved and ultimately enhance the regulations’ effectiveness. The specific research questions addressed in this study were (1) What are LA officers’ enforcement approaches to the regulations? and (2) What will enable LA officers to take an effective and constructive approach to enforcement?

## Methods

### Study design and setting

This cross-sectional study utilised qualitative research methods to examine the interplay between officers’ knowledge, experience, and their operational environment [33]. The setting for this study were LAs across England. Ethical approval for this study was granted by the University of Southampton Faculty of Medicine ethics committee (Ethics ID- 65,419.A1). The study adhered to the Declaration of Helsinki, Research Governance Framework for Health and Social Care, Data Protection Act 2018, and the Consolidated Criteria for Reporting Qualitative Studies (COREQ) recommendations [34].

### Participants

Participants were selected using purposive sampling from LAs covering the north and south of England and rural and urban areas as well as unitary, county, and borough councils. Purposive sampling was used to strategically select participants who had knowledge and/or experience of enforcement approaches within LAs. This approach was undertaken to gain exclusive insights on enforcement approaches for the upcoming regulations. Officers were recruited through contacts made with enforcement bodies, placing advertisements on trade body forums, newsletters, email groups, and trading standard meetings including introductions via participants and the government consultation documents. Further participants were recruited through snowball sampling. A diverse sample was sought to cover a range of views from officer types and officer job roles. An initial email invitation, study information sheet, and semi-structured interview guide were sent to 40 potential participants introducing the research team and providing an opportunity to ask questions before scheduling an interview. Each potential participant was followed up once or twice via email and/or phone (where available) to assess their interest. Non-responders were recorded as decliners. All participants completed a short questionnaire (providing information on their LA, job title, role, and experience) and completed a consent form (by email or verbally) prior to the interviews.

### Data collection

Data were collected over a 9-month period between August 2021 and April 2022, before implementation of the regulations in October 2022. Participants were not known to the researchers before study commencement. Interviews were held individually or in pairs. All interviews were conducted by SM and PD and recorded using MS Teams video conferencing software. Semi-structured interviews were deemed the most suitable approach to uncover participants’ lived experiences of interactions with businesses preparing to implement the novel regulations [35]. The interview schedule was based on the findings of a systematic review of related literature and finalised by the research team (Additional file 1). Participants were asked about their opinions on the regulations, how they planned to enforce them, and any potential barriers to enforcement. On average, the interviews lasted 35 min. Regular debriefs during data collection among SM, PD and CV enabled alteration of prompts to seek additional detail.

### Thematic analysis

Reflexive thematic analysis was undertaken following an inductive approach that was guided by the research questions and a constructivism philosophical paradigm which purports that individuals develop their reality using knowledge and personal experience over time [36, 37]. Each interview was transcribed verbatim by a professional transcription company. Anonymised transcripts were imported into NVIVO software (version 14) [38] for coding management. The first author led the analysis by reading the transcripts for familiarisation, making notes and coding against the research questions. The codes generated were clustered under four initial themes: (i) assessing enforcement approach, (ii) challenges with enforcement, (iii) encouraging business compliance, and (iv) enforcement support needs. The co-authors supported the analysis by reading two transcripts each, discussing the codes and reviewing the key themes. The scope of each theme was discussed in detail, with new codes merged under existing themes where appropriate or developed into a new sub-theme. SJ coded eleven transcripts to explore multiple dimensions of the data and refined themes were discussed with qualitative researchers to enhance richness. All researchers were women aged 30–60 years with expertise in public health, nutrition, food policy, psychology, and geography. The final themes and analysis story were discussed with a representative from a LA (also study participant) to further elaborate study findings.

## Results

### Participants

A total of 22 officers from five English regions participated in interviews (Table 2) comprising 13 TSOs, six EHOs, and three PHOs. Five of the 13 TSOs provided paid primary authority support to bigger retail businesses on the regulations.

**Table 2:** Officers’ regional representation and job roles

*Local authority officers (n* = *22)*	North	Central	South	London borough	Wales	Job roles
Trading standards (*n* = 13)	3	2	8	0	0	National representative/food lead (1), senior officer (7), officer (5)
Environmental health (*n* = 6)	2	1	1	1	1	National representative/food lead (1), senior officer (2), officer (3)
Public health (*n* = 3)	0	0	2	1	0	Senior officer (1), officer (2)

Six themes were identified to answer both research questions; the first three themes describe the challenges officers were facing while taking on the new regulations and their enforcement approaches, and themes 4, 5, and 6 describe contextual needs that would optimise the enforcement of the regulations. Each theme is described below, with illustrative participant quotes to provide authentic insights into the topics discussed. Interviewees are referred to using anonymised identification numbers along with their job roles.

### Research question 1: What are LA officers’ enforcement approaches to the regulations?

#### Theme 1: Food (Promotion and Placement) regulations require a new enforcement framework

##### 1a. Incompatibility with current regulatory approaches

Officers recognised that the regulations are complex and do not fit well within existing regulatory frameworks such as those for food safety, advertising, smoking, and single use plastic carrier bags. Other food policies (e.g. use by dates) allow for the relatively straightforward assessment of compliance, making it easier for officers to determine whether businesses meet regulatory standards. In contrast, the new regulations are more complex because they require officers to understand how to calculate NPM scores to determine compliance.

"Those [use by dates] are relatively straightforward for officers to get their heads round and know exactly what they’re doing. Food labelling in a similar fashion. So, I think it is unlikely that there will be a lot of proactive enforcement of the  regulations." -13004, TSO

"There’s a lot of complexity behind it. Understanding nutrient profiles, which foods are in or out. And you know, needs an in-depth level of understanding." - 13003, TSO

Some participants were familiar with the NPM which defines whether a product is HFSS, having used it to enforce the LA unhealthy food advertising ban in London boroughs on Transport for London (TfL).

"So, we do use NPM for that [TfL advertising policy], to work out what we class as foods and drinks that are high in fat, sugar, and salt. So, we have some experience of those kinds of borderline products." -13037, PHO

However, there was no comparable framework available for evaluating businesses falling under the remit of the regulations. Unlike the established Sunday trading laws, which apply to stores larger than 3,000 square feet, the new regulations set a lower threshold of 2,000 square feet. This difference requires officers to adopt new rules that do not match existing infrastructure and methods. Consequently, officers believed they would need to identify ways to obtain and interpret data on store dimensions, layouts, and employee counts.

"It might have been easier and more straight forward to have relied on the Sunday Trading Rules. Everybody knows stores that are affected and aren’t affected, and it doesn’t require any kind of calculation or justification from the enforcing officer." - 13004, TSO

"… I mean, one of the greatest difficulties for us will be ascertaining actually how many people work in the business." -13036, EHO

##### 1b. Variability in officers’ enforcement practices

Participants reported that whether a TSO or EHO will be enforcing these regulations will be determined at the LA level. These groups of officers possess and have access to different skills, knowledge and training, and evaluation of compliance will likely depend on their  discipline-specific standard procedures and experiences with different regulations. This situation will lead to diverse practices across different regions and LA types.

"We [trading standards] go out and do Food Standards inspection. Environmental health are very much hygiene and safety of food. If they were adding this to their inspection, I think that it would be, one of the lesser important things on their inspection. For us, it would just become a routine check, but yeah, there will be differences up and down the country certainly." -13023, TSO

Moreover, the complexity of the regulations and ambiguity of the regulation guidance was perceived to be contributing to different enforcement practices across authorities. Officers in business support roles were finding it challenging to offer consistent advice to businesses. They anticipated discrepancies in each enforcement officer’s ability to determine the specifics of included/excluded products, businesses, and in-store locations resulting in varying levels of implementation and enforcement.

"It’'s so ambiguous. What is an end of an aisle? How many bargain buckets, do you need in a row for that to then constitute as an aisle and that’s going to be hard as a primary authority officer because you will have someone in Cornwall saying no six in a line, I know that that’'s aisle, but you might have an officer in Lincolnshire saying no, they are just buckets, they are fine." -13029, TSO

##### 1c. Compliance is difficult to assess

Officers also felt that assessing compliance was a time-consuming and arduous task because each individual store will need assessment at three levels: in-scope business (depending on store size and number of employees), in-scope locations within stores, and in-scope products at those locations. Conducting these assessments accurately will be extremely time consuming given that officers also have enforcement responsibilities for other regulations.

"It requires additional work to establish whether or not a product falls within the regulations and whether or not any particular store falls within those regulations. Plus, each store will have to have an individual assessment as to, the position of the entrances, exits, aisles, etc. It means it’s going to be one where for any enforcer to take action, they would need to do quite a lot of assessment work." -13004, TSO

#### Theme 2: A light touch approach to these regulations

##### 2a. Insufficient capacity for active enforcement

There was a strong agreement among officers about resources and workforce constraints across LAs. Due to limited resources, officers do not conduct routine inspections of retail outlets. Instead, their enforcement actions are in response to complaints and intelligence received from areas of concern.

For food safety regulations, officers rank businesses as high- or low-risk based on Food Standards Agency’s risk matrix (which includes business size, risk to marketplace, product that poses risk, primary authority partnership, etc.) and visit premises based on their position on the risk matrix. High risk businesses may be visited every 2 to 6 months, whereas low-risk businesses tend to be visited once every 2 years. With current resource and staff shortages, officers predicted it will be incredibly difficult to enforce these complex regulations.

"We have lost three really good food officers in the space of a couple months, and we haven’t been able to fill their posts. There’s a shortage in resources. Officers aren’t going out during routine inspections. Everything has to be intel led, so you’re not getting the people on the floor. And it’s not an easy peasy regulation." -13029, TSO

Officers noted that the COVID19 pandemic had required reassigning staff to manage pandemic-related activities. Therefore, at the time of interviews, there had not been enough time and resources for officers to learn about the regulations and prepare for their enforcement.

"All of the workforce that would have been doing that work, I would say, probably 80% have been redeployed to fight COVID." -13001, EHO

##### 2b. The regulations have a low priority

Officers must abide by an enforcement code that is based on proportionality of risk which prioritises resources on regulations addressing the highest health and safety risks, such as, allergens, food safety, knife crime, and finance scams. Officers discussed avoiding situations where their LA could be challenged by businesses, especially if their notices are overturned based on the proportionality of risk and the public interest test. Other regulations addressing high risk would take precedence over these regulations because they involve immediate and clear threats to public health, necessitating urgent and strict actions. In contrast, the regulations do not target immediate health risks but rather focus on managing long-term health impacts of obesity and therefore are unlikely to be prioritised.

"And even within Trading Standards, obviously allergens are high (priority), product safety, electrical safety, imports of goods through the docks where it’s gonna be high. So, if there’s a limit on resources, that’s where it’s gonna go, rather than things that are sort of, isn’t gonna kill somebody tomorrow, to be perfectly frank." -13009, TSO

##### 2.c The process of issuing notices is burdensome

Some officers viewed notices as an effective deterrent because the possibility of facing fines can strongly discourage businesses from violating regulations. Many other participants expressed concerns about the practical challenges associated with fixed-term penalty notices including the time and cost associated with completing the necessary paperwork and pursuing payment of fines. Consequently, many officers are likely to opt for alternative approaches.

"I know fixed penalty notices are an absolute nightmare for us.... our experience of COVID fixed penalty notices, chasing non-payment and all of the paperwork that goes with that is just… Oh, it’s enormous, and the cost for the authority is huge and so there may be sort of a move to try and avoid doing fixed penalty notices." -13023, TSO

Officers also described that the *Proceeds of Crime Act 2002* act allows for money collected via fines to be used by the LAs for public benefit. The fines and confiscated assets from operations against illicit tobacco trade can typically result in substantial financial gains for LAs that they could reinvest into the community to support various public services. Comparatively, an FMP of £2500 for non-compliance with these regulations was perceived as a minor tool and would not generate significant revenue for LAs.

"If I had a choice between going and busting my local Retailer 14 for having too many chocolates, or breaking up an illicit tobacco smuggling operation, it makes financial sense and health sense to do the latter, doesn’t it?" -13040, PHO

Officers expressed concern that businesses might perceive that fines associated with fixed notices as an attempt by LAs to extract money from them, potentially straining their relationships with businesses. This would further increase difficulties to conduct compliance checks as businesses may become less cooperative and officers may face difficulty accessing their premises.

"I actually think that some of the retailers, um, will think it’s [FMP] a bit like a cash cow for the LAs." -13016, EHO

#### Theme 3: Navigating the relationships with local businesses

##### 3a.Proving non-compliance could be difficult and may cause tension

Officers described that businesses are not required to provide evidence or justification for their compliance with regulations. Instead, the officers are tasked with making judgments on whether a particular area of the business’s premises is compliant or not. This situation can lead to lengthy and contentious discussions between the officer and the business. Such drawn-out interactions can impact relationships between the business and the officers and hinder the effectiveness of enforcement efforts.

"They [businesses] don’t have to prove anything, it’s all on the officer who then says, 'Well, I think you’re wrong.' It could be a long protracted discussion with somebody who says, 'Well, no it’s not. I’m small. I’m bigger. I’m not using the entrance area.' 'Yes, you are.' You know that sort of drags on." -13003, TSO

Officers expressed concern that many businesses might initially make changes to comply with regulations but then revert to their usual practice of prominently displaying HFSS products in high footfall locations until the next visit from enforcement officers. This behaviour would pose a great challenge for officers tasked with enforcing the regulations.

"The businesses that I know, you tell them to move the stuff, they’d move it. You could go in and six months’ time it’s right back by the till again. How many times does a local officer say well I told you this last time and you’ve moved it back?" -13016, EHO

##### 3b. Unique relationships between businesses and primary authorities

Officers anticipated that larger businesses would likely be compliant and low-risk due to their primary authority partnership, which provides them with access to regulatory advice, legal protection, and assistance with implementing regulatory changes. Consequently, officers expected to conduct fewer inspections of bigger, chain stores and focusing their attention on medium and smaller businesses. They believed that smaller retailers and franchisee stores were less likely to be familiar with the regulations and more likely to encounter challenges in complying because they do not have the resources of the larger chain stores. Officers recognised the need to identify approaches that could influence practice in smaller stores effectively, and this approach would be more light touch.

"Those with a primary authority will be putting plans in place and will pretty much self-regulate. Those without a primary authority won’t know it exists…. some will try and respond to it, I think a lot will struggle, they [smaller businesses] will need more support in doing this." - 13039, EHO

##### 3c. Supportive enforcement strategies will be used

Officers expressed concerns that if businesses believe the regulations will negatively affect their profits from HFSS foods, they are likely to resist complying. The regulations are perceived as addressing low health risk issues. Therefore, local councillors, who want to ensure businesses in their areas feel supported, may be reluctant to endorse the implementation and enforcement of the regulations.

"I think the tension comes, and this happened with the advertising policy as well, if you’re getting pushback from businesses because they feel that it’s impact on their revenue, you know, if our councillors are hearing that, then that can be a bit of a challenge to the health and wellbeing agenda." -13037, PHO

Officers acknowledged that the route to achieving compliance with the regulations is through supporting businesses. All officers affirmed that they would employ informal enforcement techniques, such as engaging with businesses to collaboratively find solutions, raising awareness, offering advice, and negotiating, rather than taking punitive measures such as issuing improvement or FMP.

"We should be saying to them, 'Well, it’s your responsibility to comply. These are the rules, how are you going to do it?' And then, engage with them around if it will work or not. If it gets to a point where they just kinda go, 'Well, we just can’t comply, or we aren’t going to comply,' So the idea is to get them back into compliance without having to head down slightly more formal improvement notice route. I think the enforcement will be kind of low key engage and improvement notice is very much a kind of last resort." -13003, TSO

### Research question 2: What will enable LA officers to take an effective and constructive approach to enforcement?

#### Theme 4: Further leadership from national government

##### 4a. Set indicators and provide adequate enforcement funding

Officers believed that the central government should enhance enforcement efforts by providing funding, establishing performance indicators for local governments, and systematically measuring these indicators over time. Similar measures proved effective in previous tobacco legislation, ensuring accountability and proactive enforcement. Many officers stated that grants to regional trading standards liaison groups can allow LAs to complete targeted project work and conduct visits to raise awareness about the regulations and encourage compliance. Having allocated funding will enable support to be directed towards businesses who do not have paid primary authority support.

"...sometimes what’s useful is when new regulations are introduced to have some sort of grant so that officers have some funding maybe to bring in some temporary resource, you know, to do some work initially." -13001, EHO

Officers felt that government could strengthen implementation of the regulations at a local level by setting and assessing enforcement indicators. Establishing clear indicators can provide accountability for LAs and increase the likelihood that regulations will be enforced.

"So, I would say they probably would be better off making it an indicator, making it measurable. Then you can link it to other indicators, and then local policy decision makers, like the Public Health Directors can then use it to set local policy. And then that’ll drive the use of that indicator at the local level." -13010, EHO

##### 4b. Training and tools for enforcement officers

In addition to funding and accountability indicators, officers asked for the provision of central training to increase their confidence in interpreting the regulations guidance and assessing business non-compliance. They suggested that training could be provided virtually so they can train at a time that suits them.

"If they could put some training packages together, particularly as the regulation guidance is issued. So that officers get that from a central point, and we’re all starting from the same understanding of what the regulations actually mean." - 13033, TSO

Many officers emphasised the necessity of assistance in accurately scoring in-scope products, including cultural foods. They asked for a central NPM score calculator that is accessible to both businesses and officers, aiming to promote consistency, prevent confusion, and offer technical guidance on products falling within relevant categories. Such a tool would help to ensure officers use their time and resources efficiently. Additionally, officers expressed a desire for ongoing support from central government and refinement of the guidance to address practical issues.

"Is the government better off actually speaking to all those sorts of interested parties and honing it [NPM calculator] down so that people are giving consistent results what if one says it’s in and the other says it’s out or whatever?" -13003, TSO

"If an issue escalates, it can be really helpful for a regional group, to put questions to the Department of Health and Social Care, and any other sort of central government body, that kind of ongoing support is gonna be really helpful." - 13000, TSO

##### 4.c. Redistributing responsibility across stakeholders to increase efficiency

Officers felt that there is disparity in the burden on retailers for scoring products for NPM scores, especially the smaller stores who have limited resources. They suggested that the government should mandate manufacturers to provide NPM scores for their products and continually update them following reformulations. This directive will help share the responsibility for determining product NPM scores and increase consistency.

"I think it needs to be spelt out in the legislation that the responsibility of making a decision about whether something is in scope or not in scope should fall to the manufacturer and this information should be freely available upon request, like the safety data sheets have to be available upon request from a retailer." - 13029, TSO

Officers believed they should have easy access to information about the store size; if national government made it a legal requirement for businesses or primary authorities to provide information about the store size and layout zones, it could make enforcement more efficient.

"Maybe that’s a central government thing and/or primary authority, if they have stores that are straddling the threshold then maybe we can be provided a list of which stores are in and which stores are out. So that LA [officers] don’t have to go chasing round for those details." - 13003, TSO

#### Theme 5: Regional and local actions to support effective enforcement

##### 5a. Cross-departmental collaboration

Officers highlighted the benefits of proactively instigating linkages within each LA by promoting and supporting cross-departmental activity between public health, officers in business support roles, TSOs, and EHOs. These supportive partnerships could facilitate improved consistency across regions in commissioning of projects for implementing and enforcing food policies at local level.

"We do have a good food retail like meeting, it’s a subgroup of our Healthy Weight Taskforce. So Environmental Health is represented, our business support team is there, our Public Health team is there. And we’ve got a social enterprise in our borough, that works specifically around food, that often gets commissioned by the Council." -13037, PHO

Engagement between public health and other LA teams could also help draw potential scenarios of breaches and methods to assess compliance. Officers believed that for consistent enforcement, discussion among officers in business support roles and enforcement roles can help develop a consensus and best practice guidance that is acceptable under the law. This guidance could be applied consistently across store types and would also be helpful for smaller stores that lie outside of primary authority relationships.

"We’re going to have a combined meeting of the major supermarkets. It may well happen across the other primary authority groupings where we have meetings just to sort of talk about the practicalities of it in a collective way, so that there aren’t inconsistencies and Retailer 23 don’t do something completely different from what Retailer 4 do or whatever."-13003, TSO

##### 5b. Raising the regulations’ profile within each LA

Officers felt that incorporating the regulations into Joint Strategic Needs Assessments (JSNA) within LAs would help elevate the importance of the regulations’ enforcement. JSNAs are comprehensive assessments conducted by LAs to understand the health needs and priorities of their communities. Increasing prominence of these regulations would help secure buy-ins from councillors and public health directors which in turn could help increase priority for enforcement and resource allocation with LAs.

"And I think it’s something that we could try and push to get the management and the councillors on board with. But it’s got to come from above me, I’m afraid, to have somebody to have the will to say, 'This is important.' Because as it is, I just don’t think it’ll get done." -13009, TSO

#### Theme 6: Building societal support to change marketing norms

##### 6a. Create an understanding of marketing strategies among citizens

Officers highlighted the need to raise awareness about the impact of extensive marketing of unhealthy products on citizens’ food choices. This increased awareness could help generate the support needed for successful implementation of the promotion component of the regulations. They further emphasised that simply having the regulations in place is not enough for effective enforcement. Similar to tobacco regulation, there needs to be a broader understanding and acceptance among consumers regarding the issues with how food is marketed and regulated.

"This is a culture change. So, all these things take their time but there was a fair wind behind the no smoking. So, we didn’t have riots on the streets, and it happened. But if there isn’t a fair wind behind it [the regulations] and the public aren’t keen on it, it may just wither and die because nobody’s really interested in it." -13003, TSO

##### 6b. Increase public pressure to support regulatory efforts

Officers discussed that engaging the public through campaigns could empower citizens to challenge unhealthy product placement in retail settings. When consumers notice violations of regulations, such as unhealthy product placement, they could report these issues to authorities or question supermarkets, thereby aiding enforcement efforts. This would over time normalise reduced promotions of unhealthy foods ultimately decreasing the need for proactive enforcement.

"You know, public pressure can be really important and when you’re talking about compliance, consumers can be a very strong lobbying force and they do like to be the eyes and ears and I think that’s gonna be very valuable and important on this."- 13001, EHO

## Discussion

### Principal findings

Our analysis revealed that enforcement officers perceive the regulations to be complex, onerous, and addressing a low health risk issue in comparison to immediate risks to health such as allergens. These regulations do not fit easily with their current enforcement approaches; thus, motivation to enforce these regulations is low. A light touch enforcement approach will be adopted due to structural challenges including resource constraints, prioritisation of other regulations that address more critical health risks, and avoidance of the arduous task of issuing FMP. Officers were also mindful about fostering constructive relationships with local businesses for local economic and enforcement reasons and will therefore encourage compliance through supportive approaches. Enforcement of the regulations is highly likely to be patchy and ineffective unless further funding is provided by national government to support active enforcement practices. Investing in enforcement of these regulations in the initial years is important to ensure consistent compliance by businesses and minimise unintended impacts.

Our findings suggest that the national government can facilitate consistent and efficient enforcement by ensuring (i) manufacturers disclose product NPM scores regularly, particularly following product reformulation; (ii) businesses or primary authorities provide store size information to all LA enforcement teams; and (iii) funding is provided and enforcement targets are set. At the local levels, strengthening linkages between public health, business support, trading standards, and environmental health teams could embolden local councillors to support incorporation of the regulations into JSNA and encourage cross-departmental collaboration. Furthermore, officers expressed the need to change societal norms where citizens believe that marketing of unhealthy foods is harmful to their families and voice the need for healthful retail environments.

### Comparison with previous literature

In 2019, TfL introduced restrictions on junk food advertisements on their public transport networks in London. These regulations highlighted the practical challenges for using the NPM model to score products in terms of health and led to the subsequent incorporation of an exceptions process to simplify enforcement [39]. Our findings illustrate similar challenges for LA officers in determining in-scope products and in-scope stores. Clear, accessible resources are needed to reduce the time required for businesses to implement the regulations and for LA officers to practice accurate enforcement.

Previous research shows that EHOs apply learnings and experiences from the range of regulations within their portfolio to new regulations which increases their speed at making enforcement decisions [40]. This previous research is consistent with reports in our study that enforcement practices for the regulations are likely to differ between TSOs and EHOs because they will draw upon their previous experiences of enforcing very different sets of regulations. The distinct focus of EHOs on food safety and TSOs on consumer protection and fair trade practices also influences the way they approach enforcement actions. This finding highlights the importance of the provision by central government of accredited training to ensure officers with different professional backgrounds and working in different regions obtain a common interpretation of regulations and enforcement practices to ensure consistency across England and maximise potential health benefits.

While enforcement is necessary for all regulations, the reality of resource constraints and prioritisation pressures can lead officers to focus on higher-risk areas. This focus can create a perception among both officers and businesses that low-risk regulations are less urgent, potentially leading to a less active approach to enforcement. Officers participating in this study described their intentions to use light touch enforcement approach, offering support and reminders of the regulations rather than using monetary penalties to evoke compliance. Many cited that this light touch approach was necessary due to resource constraints, regulation complexity, and relative risk of the regulations compared to other higher health risk regulations (i.e. food safety and food allergens). Previous research suggests that this lighter touch enforcement is also consistent with other existing nutrition-related regulations such as health claim labelling [41], further illustrating the perceived lower importance of nutrition-related regulations among officers. Despite these challenges, business adherence and compliance assessment of all regulations remains crucial.

### Policy and research implications

While this study focuses on the enforcement of regulations aimed at restricting the promotion of unhealthy foods, it is important to recognise that obesity is a multifactorial issue. Participants’ perspectives highlighted that nutrition related policies appear to address a relatively low health risk issue (e.g., obesity) in comparison to other immediate risk factors (e.g., allergens) which they must enforce. Therefore, the regulations are considered a low priority within some LAs. Additionally, the complexity and lack of adequate enforcement funding for these regulations further reduces their perceived level of priority*.* With the vast costs of obesity, including the significant burden on the NHS and societal workforce productivity losses amounting to £98 billion per year [42], nutrition-related food policies should be given greater prominence in regulation enforcement. Lessons from Tobacco Control Plan indicate greater potential for impact when regulations are appropriately enforced [43]. The UK central government provides public health grants to LAs to enable the implementation of the Tobacco Control Plan and related enforcement activities for England [44]. Similar funding pots should be provided for the regulations to provide adequate resourcing for enforcement. In addition to adequately resourcing regulatory initiatives, building public support for regulatory action can significantly enhance policy effectiveness, as demonstrated by the success of tobacco control policies [45]. The framing of message relating to the protection of non-smokers from the consequences of second-hand smoke helped to transform self-regulation into government legislation [45]. With growing public acceptance of nutrition-related policies such as sugar taxes, there is an opportunity for a public discussion about the harms caused by unhealthy food marketing practices to harness societal support for strong enforcement [46, 47]. Increased consumer awareness of pervasive marketing strategies could also help to garner support for promotions component of the regulations which has been delayed until October 2025 due to the cost-of-living crisis [48].

Our research underscores the clear gap between the government’s implementation of the regulations as a crucial component of its obesity strategy while simultaneously not adequately resourcing its enforcement. A total of £179,000 has been offered across 317 LAs in England to support enforcement of the regulations in its first year and reduced to £102,000 in the second year [49]. Three months after the legislation was implemented, a Freedom of Information request by the trade magazine Convenience Store revealed that only two LAs had recorded any violations, with no improvement notices issued [50]. Additionally, over a year after the regulations came into effect, a report by the Obesity Health Alliance indicated that only two out of 32 officers had conducted inspections under these regulations, and no improvement notices had been issued to businesses [51]. With many LAs across England facing significant financial difficulties, a risk-based enforcement model is being applied where compliance is not regularly evaluated but rather addressed reactively in response to complaints. If there are no complaints, compliance may not be thoroughly assessed and instead is reliant on the expectation that businesses will uphold their regulatory responsibilities resulting in self-regulation [52]. It has been widely documented in the literature that self-regulation commitments of food industry are proven to not work [16, 53]. While this approach is resource efficient for government, it does not create a fair playing field across businesses. Revenue is the main priority for businesses and compliance will be low if there is no benefit to them and there are no serious repercussions for non-compliance [54]. Future research should investigate the cost and time implications of active enforcement of the regulations across business types to provide government with accurate financial predictions that will enable the level playing field desired by businesses and health advocates [55, 56]. Furthermore, ways of offering benefits to compliant businesses, rewarding positive practices, and refining the FMP to be proportionate to the size of business should also be assessed. With the Pure Food and Drugs Act in the United States, for example, the threat of fines was ineffective, so enforcement was obtained by offering benefits to compliant firms through product quality certification which was enticing for businesses [57].

The risk posed by unhealthy retail food environments in not immediate, in contrast to food safety scares, and therefore perception of low health risk from unhealthy food consumption weakens the action that the officers might take. However, obesity risk is at a population level and has implications for a much larger number of citizens over a longer term. Public health teams can continue to play a vital role promoting awareness of the regulations and highlighting cases of non-compliance to catalyse enforcement efforts and maximise public support for nutrition-related food policies.

### Strengths and limitations

A strength of our study is that the participating officers were drawn from different LAs across England and the findings represent varied perspectives from a range of different types of LAs and departments. The findings have important implications for enabling effective enforcement of these landmark regulations and offer an important contribution to the limited literature on food policy enforcement practices more widely. During data collection, the researchers’ backgrounds as a public health nutritionist and psychologist facilitated effective engagement with LAs. During the analysis phase, the first author regularly consulted with co-authors to ensure the analysis adhered to rigorous qualitative research methodology.

Our findings captured several aspects that will influence the enforcement of the regulations. However, qualitative findings are often context-specific and may not be universally applicable. Consequently, other factors may serve as levers or hindrances of enforcement activities in different countries or contexts. This study reflects only the pre-implementation position of the officers, and it is possible that the officers’ views and practices on these regulations may have shifted post-implementation. Collecting data post-implementation would have further enhanced these insights however it was not possible within the resources and timeframe of this study. Nevertheless, this study sets a baseline for following up LA officers to assess their enforcement strategies post-regulation implementation. Although efforts were made to ensure a mix of views from TSOs, EHOs, and PHOs from LAs across England, participants largely self-selected to participate through advertisements via Chartered Trading Standards Institute (CTSI) and Chartered Institute of Environmental Health (CIEH) networks, food groups, or by emailing LAs. Officers who did not choose to participate may hold different views or may have different levels of knowledge about the regulations. It is also possible that changes in government ministers and their priorities, which led to a delay in implementation of the promotion component of the regulations, may have influenced enforcement approaches towards the regulations.

## Conclusions

Officers perceive the regulations to be complex and onerous and due to time and resource constraints expect to adopt a light touch approach to enforcement. This light touch approach may lead to reduced and uneven compliance rates with the regulations. To optimise enforcement efforts, both structural challenges and regulation complexity needs to be addressed. A consistent and coordinated strategy is imperative which will require (i) increased leadership from the central government including funding, training, and clearer guidance on what constitutes in-scope businesses and products; (ii) collaborative efforts and prioritisation across departments at LA and regional levels; and (iii) a concerted effort to shift societal norms through consumer engagement. These regulations are pioneering and progressive internationally and may positively influence population diet. Its success hinges on robust enforcement and compliance, necessitating the provision of adequate financial and technical resources for LAs, which should be integral to the implementation of these regulations.

## Supplementary Information

Additional file 1. Question guide – Local authority officers.

## Data Availability

The data were collected by the research team for the purpose of this study. The data can made available upon reasonable request to the corresponding author pending approval.

## Abbreviations

HFSS: High fat, salt, or sugar
LA: Local authority
EHOs: Environmental health officers
TSOs: Trading standards officers
NPM: Nutrient profiling model
FMP: Fixed monetary penalty
PHOs: Public health officers
COREQ: Consolidated Criteria for Reporting Qualitative Studies
TfL: Transport for London
CTSI: Chartered Trading Standards Institute
CIEH: Chartered Institute of Environmental Health
