# Supplementary material for: “If government is saying the regulations are important, they should be putting in funding to back it up.”- An in-depth analysis of local authority officers’ perspectives of the Food (Promotion and Placement) (England) Regulations 2021
Source: BMC Med. 2024 Nov 6;22:514. doi: 10.1186/s12916-024-03720-5 (PMC11539601; doi:10.1186/s12916-024-03720-5)
Supplement: Supplementary file 1 — Additional file 1. Question guide – Local authority officers. [file 12916_2024_3720_MOESM1_ESM.docx]

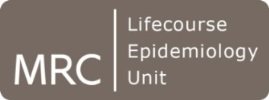

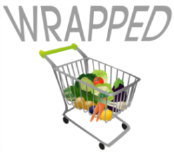


# MRC Lifecourse Epidemiology Unit

Southampton General Hospital

# Southampton S016 6YD

Telephone: 023 8120 4186


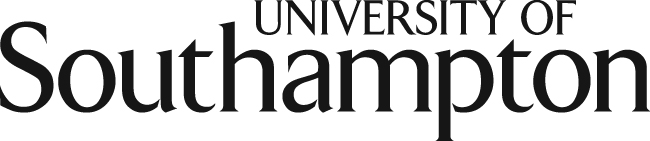


d d m m y y

**Local authority:**  **Date**: T**ime:**

**Question guide – Local authority officers**

**Introduction**

Hello, I’m a *[name]* from the University of Southampton. We are conducting research to understand your **views on the upcoming legislation restricting promotion and placement of unhealthy foods** in retail outlets.

For this research we will be asking some questions to understand your views as a local authority officer. We would like to record the meeting with your permission. The recording will be typed up and read by the research team and any names will be removed from the written version.

We encourage you to have your cameras on during the interview, but we understand if you would prefer to have them off. Your names will not be reported at any point. So please be assured that your contribution in the interview will remain anonymous and confidential in written reports. The interview will last approximately 30-45 minutes. You can choose not to answer questions. If you wish to leave the discussion at any point, you are of course able to do that. We really value your contribution as your views will bring an important angle in terms of legislation implementation and enforcement.

**Prompting questions**

1. **What are your opinions of the legislation for restricting the promotion and placement of unhealthy foods (high fat, sugar and salt products) in retail store outlets?**

*Prompt*

- *Opinions about the need for the legislation*
- *Main benefits and/or concerns*

1. **How clear is the upcoming legislation?**
   - in-scope products
   - in-scope businesses
   - in-scope areas within retail stores
   - *What is needed to make the legislation clearer?*
2. **How much of a priority do you believe the legislation will have in your local authority?**
   - Barriers to prioritisation
     1. Solutions to barriers
   - What has happened in the past to increase prioritisation of the new legislation?
3. **What, if any, support would be helpful for the enforcement of this legislation?**

*Prompt*

- *Financial support/equipment from central*
- *Guidance/case studies from Local Government Association or other professional bodies*

1. **How could the enforcement of this legislation be accommodated within the existing workload of your team?**

*Prompts*

- *Capacity concerns for ongoing implementation of the legislation enforcement*
- *Additional resources to assist with enforcement (e.g. from professional bodies, central government – e.g. toolkit for enforcement, digital app etc)*

1. **(If some of the barriers related to prioritisation could be solved…)**

**How would your local authority go about enforcing this legislation? *Prompts***

- *Whose role would it be?*
  - *Links made with other authorities to collectively approach enforcement of this legislation*
- *What tools/information will be used to assess compliance with the proposed definitions?*
- *Enforcement approach for smaller and larger outlets and for online outlets*
- *How will non-compliance be dealt with?*
- *How will fines be issued.*

1. **How might strategies for enforcement differ in other local authorities?**
   - Any suggestions to help ensure legislation is consistently enforced across different regions?
2. **How do you think businesses are responding to this intended legislation?**

*Prompts*

- *Impact on retailers?*
- *(if not covered above) Differences in compliance by outlet type and size*

1. **What impact do you think the legislation may have on your local community?**

*Prompts*

- *Impact on consumers, society and government*

1. **More generally, who do you think is responsible for supporting healthy eating?**

*Prompt*

*Responsibility of retailers/food industry, government or individuals*

**End with:**

- Any last comments or thoughts that we haven’t yet discussed?
- Thank you for your time.
